# Maternal and neonatal outcome of births planned in alongside midwifery units: a cohort study from a tertiary center in Germany

**DOI:** 10.1186/s12884-020-02962-4

**Published:** 2020-05-06

**Authors:** Waltraut M. Merz, Laura Tascon-Padron, Marie-Therese Puth, Andrea Heep, Sophia L. Tietjen, Matthias Schmid, Ulrich Gembruch

**Affiliations:** 1 Department of Obstetrics and Prenatal Medicine, University Hospital Bonn, Venusberg-Campus 1, 53127 Bonn, Germany; 2 Department of Medical Biometry, Informatics and Epidemiology, Faculty of Medicine, University of Bonn, Venusberg-Campus 1, 53127 Bonn, Germany

**Keywords:** Midwife-led care, Alongside midwifery unit, Low-risk pregnancy, Transfer rate, Maternal healthcare provision, Obstetric service, Mode of birth, Obstetric intervention

## Abstract

**Background:**

For healthy women entering birth after uneventful pregnancy, midwife-led models of care have the potential to reduce interventions and increase the vaginal birth rate. In Germany, 98.4% of women are giving birth in consultant-led obstetric units. Alongside midwifery units (AMU) have been established in 2003. We compared the outcome of women registered for planned birth in the AMU at our hospital with a matched group of low-risk women who gave birth in standard obstetric care during the same period of time.

**Methods:**

We used a retrospective cohort study design. The study group consisted of all women admitted to labor ward who had registered for birth in AMU from 2010 to 2017. For the control group, low-risk women were selected; additionally, matching was performed for parity. Mode of birth was chosen as primary outcome parameter for the mother. For the neonate, a composite primary outcome (5-min Apgar < 7 or umbilical cord arterial pH < 7.10 or transfer to specialist neonatal care) was defined. Secondary outcomes included epidural anesthesia, duration of the second stage of labor, episiotomy, obstetric injury, and postpartum hemorrhage. Non-inferiority was assessed, and multiple logistic regression analysis was performed.

**Results:**

Six hundred twelve women were admitted for labor in AMU, the control group consisted of 612 women giving birth in standard obstetric care. Women in the study group were on average older and had a higher body mass index (BMI); birthweight was on average 95 g higher. Non-inferiority could be established for the primary outcome parameters. Epidural anesthesia and episiotomy rates were lower, and the mean duration of the second stage of labor was shorter in the study group; second-degree perineal tears were less common, higher-order obstetric lacerations occurred more frequently.

Overall, 50.3% of women were transferred to standard obstetric care. Regression analysis revealed effects of parity, age and birthweight on the chance of transfer.

**Conclusion:**

Compared to births in our consultant-led obstetric unit, the outcome of births planned in the AMU was not inferior, and intervention rates were lower. Our results support the integration of AMU as a complementary model of care for low-risk women.

## Background

Advantages of Midwife-Led Care (MLC) models have been widely reported. For healthy women entering labor after an uneventful pregnancy, various beneficial effects have been observed. These include, among others, an increased likelihood of giving birth vaginally; a lower intervention rate, including epidural anesthesia and instrumental vaginal birth; and a shorter duration of labor [1–6]. Due to the low incidence of severe perinatal morbidity and mortality in high-income countries, less data are available with respect to the newborn.

MLC is practiced in different settings, including home births, births in freestanding midwifery units (FMU), and births in alongside midwifery units (AMU). Within these different settings, MLC may be restricted to the time of birth or may constitute a continuity of care during pregnancy, birth and postpartum. MLC may be organized as caseload MLC, where one midwife or a small group of midwives is attending to a woman throughout pregnancy, birth and postpartum, or a conventional type of MLC; the latter does not imply continuity of care by one professional. Moreover, according to the organization of the maternal healthcare system, practices in these models of care vary between countries. These pertain for example to transfer modalities to standard obstetric care in case of complications during or immediately after labor. Therefore, studies about MLC may yield different results.

In Germany, 98.4% of women are giving birth in obstetric units. Here, the care of women in labor is shared by the attending midwife and obstetrician. The remaining 1.6% of births take place at home or in FMUs. Outcome data of out-of-hospital births are collected on a voluntary base, and show a low rate of complications [7]. However, due to the low numbers, non-uniform eligibility criteria and non-standardized transfer modalities, the figures do not allow an evaluation of this model of care with regard to maternal and perinatal safety.

Supported by a team of researchers, care in AMU has been introduced in Germany in 2003 [8]. AMUs are hospital-based and located within obstetric units. Births in AMUs are offered in addition to standard obstetric care by the same team of midwives. Low-risk women who have chosen this model of care are attended to by a midwife from the team, with transfer to standard obstetric care in case of abnormalities. Continuous, one-to-one support is being provided. Since both models of care are situated within the same premises, transfer to standard obstetric care occurs without delay. Indications for transfer include, among others, maternal request for intravenous or epidural analgesia, and necessity of oxytocin augmentation.

The maternal and perinatal outcome of births in AMUs in Germany has not yet been investigated. The German birth registry [9] does not allow for an analysis of these cases, since births in AMUs are not specifically labelled. Additionally, obstetric departments with established AMUs have no legal obligation to report the outcome of births in this model of care.

The Department of Obstetrics at the University Bonn Medical School introduced an AMU in November 2009. Data of all eligible women (medical and obstetric history, details of the birth, transfer cause if applicable, and maternal and newborn outcome) are entered into a computer-assisted database, irrespective of the actual place of birth (AMU or standard obstetric care, in case of transfer during or immediately after birth).

The aims of our study were (i) to compare the maternal and perinatal outcome of women registered for birth in the AMU at our hospital, a level three university hospital department, with births of low-risk women in standard obstetric care, occurring in our unit during the same period of time; and (ii) to investigate causes for and outcome of births of women who were transferred from AMU to standard obstetric care during or immediately after birth.

## Methods

Women planning to give birth in AMU were assessed by a midwife from the team during an antenatal visit in late pregnancy. A checklist which had been developed jointly by the team of obstetricians and midwives was applied. Likewise, a checklist with criteria for transfer to standard obstetric care during labor was in place.

A retrospective cohort study design was used. The study group consisted of all women admitted to the labor ward at the Department of Obstetrics, University Hospital Bonn, who had registered for birth in the AMU between January 2010 and December 2017. Only singleton term cephalic pregnancies were included. Exclusion criteria were as follows:

(1) Medical history: preexisting medical condition (e.g. type 1 diabetes, cardiac disease); body mass index (BMI) > 35 kg/m^2^.

(2) Obstetric history: complications during previous deliveries (e.g. shoulder dystocia; postpartum hemorrhage); previous cesarean section (CS).

(3) Complications during the ongoing pregnancy: maternal complications (e.g. pregnancy-induced hypertension / preeclampsia; insulin-dependent gestational diabetes); amniotic fluid abnormalities (oligo- or polyhydramnios).

Indications for transfer to standard obstetric care during birth included delayed first or second stage; oxytocin augmentation; fetal heart rate (FHR) abnormalities; maternal pyrexia; and request for i.v. opioid or epidural analgesia. Indications for transfer to standard obstetric care immediately after labor included postpartum hemorrhage and higher-degree obstetric lacerations.

The control group consisted of women who would have been eligible for birth in the AMU due to their low-risk profile, but who gave birth in standard obstetric care during the same period of time.

Women for the control group were selected as follows: For each woman in the study group, the subsequent birth in the delivery book was chosen if it fulfilled the eligibility criteria for care in the AMU (see above). Additionally, matching of cases and controls according to parity (nulliparous and parous) was performed.

Not all eligibility and exclusion criteria are mentioned in the delivery book. A detailed assessment including medical and obstetric history as well as abnormalities during the course of the pregnancy of each case identified in the delivery book was therefore performed. For this purpose the electronic departmental database (Viewpoint, GE Healthcare GmbH, Solingen, Germany) was used. Here, each woman is allocated a unique number, and each pregnancy is entered as a separate case. The database contains all medical, prenatal and obstetric data. In case eligibility was confirmed the woman was finally entered into the control group. Otherwise, the subsequent birth in the delivery book was identified and the procedure repeated.

The study was approved by the Ethics Committee of the University Bonn Medical School (registration number 254/18). Women in the study group gave their written consent for inclusion into the registry at the time of consent for birth in the AMU.

For the mother, mode of birth was chosen as primary outcome. For the newborn, a composite outcome of 5-min Apgar score < 7 and / or umbilical cord arterial pH < 7.10 and /or unplanned transfer to specialist neonatal care was defined. For the primary outcomes, non-inferiority of care in the AMU to standard obstetric care was calculated. Additionally, the following secondary outcomes were defined: For the mother, duration of the second stage of labor; epidural analgesia; postpartum hemorrhage; obstetric injury; and episiotomy. For the newborn, Apgar scores at one, five and 10 min; and Base Excess (BE) in the umbilical cord artery. In case of transfer to standard obstetric care, causes for transfer were recorded; likewise, indications for operative vaginal birth or emergency CS were noted.

### Statistical methods

The analysis was performed on an intention to treat approach. Descriptive analyses were performed to examine the basic characteristics of the study and control groups. Differences between the matched groups were evaluated using McNemar’s tests (for categorical variables) and paired sample t tests (for continuous variables). The risk difference between paired proportions of each primary outcome variable (measured in percent) together with the 95% confidence interval (CI) was determined. As the intent of our analysis was to demonstrate that the safety of AMU care with respect to the primary outcomes is not worse than the standard obstetric care by more than a small amount, we assessed non-inferiority using the risk differences between both models of care. Non-inferiority of AMUs to standard obstetric care was declared if the lower bound of the confidence interval of the difference did not fall below the non-inferiority margin of − 2%. Subsequently, generalized linear mixed-effects models were used to assess the association between the type of care and the mode of birth (operative vaginal/CS versus spontaneous birth) or the neonatal composite outcome (yes versus no). We adjusted for parity, maternal age, maternal BMI and birthweight. Further multiple logistic regression analysis was used to identify factors for a transfer to standard obstetric care during or immediately after birth in the study group. Analyses were carried out using R (version 3.5.2) and SAS® Software (version 9.4, SAS Institute Inc. Cary, NC, USA).

## Results

During the study period, 612 women were admitted for labor in the AMU. Likewise, the control group consisted of 612 low-risk women, matched for parity, who gave birth in standard obstetric care.

(a) Outcome of births in the study and control group.

Table 1 summarizes maternal, obstetric, and neonatal data of the study and control group. The women planning to give birth in AMC were on average older (mean 32.9 years, SD 4.4 versus mean 32.1 years, SD 5.1). The rate of overweight or obese women (BMI 25.0–35.0 kg/m^2^) was higher in the study group (68.8% versus 32.0%). Six women with BMI > 35 kg/m^2^ (maximum BMI 36.8 kg/m^2^) had been erroneously recruited in the study group; they were included in the analysis.

**Table 1:** Basic characteristics of the study and control group (*N* = 1224)

	Study group (*n* = 612)	Control group (*n* = 612)	*p* value
Age (years), mean (SD)	32.9 (4.4)	32.1 (5.1)	0.003
BMI (kg/m^2^) ≥ 25, n (%)	421 (68.8)	196 (32.0)	< 0.001
Mode of birth, n (%)			0.101
Spontaneous	517 (84.5)	502 (82.0)	
Instrumental vaginal	38 (6.2)	58 (9.5)	
Cesarean	57 (9.3)	52 (8.5)	
Epidural anesthesia, n (%)	117 (19.1)	252 (41.2)	< 0.001
Duration, second stage of labor^a^ (min), mean (SD)	47.4 (54.1)	55.6 (59.5)	0.002^b^
Episiotomy^a^, n (%)	26 (4.7)	48 (8.6)	0.066
Obstetric injury^a^, n (%)			0.007
First degree perineal laceration, labia laceration	149 (26.8)	119 (21.2)	
Second degree perineal laceration, vaginal or clitoral laceration	191 (34.4)	260 (46.4)	
Third or fourth degree perineal laceration, cervical laceration	13 (2.3)	5 (0.9)	
None	202 (36.4)	176 (31.4)	
Postpartum hemorrhage, n (%)	43 (7.0)	55 (9.0)	0.246
Birthweight (gram), mean (SD)	3561.0 (427.3)	3466.3 (422.5)	< 0.001
5-min APGAR score < 7, n (%)	3 (0.5)	2 (0.3)	1.0
Umbilical cord arterial pH < 7.10, n (%)	8 (1.3)	16 (2.6)	0.153
Missing	12 (2.0)	3 (0.5)	
Transfer to specialist neonatal care, n (%)	5 (0.8)	9 (1.5)	0.386

*BMI* Body mass index, *SD* Standard deviation

^a^Missing for pairs with mode of birth = cesarean

^b^Wilcoxon signed-rank test

There was no difference in the primary maternal outcome. The analysis of secondary maternal outcomes yielded the following results (see Table 1): The epidural anesthesia rate was significantly lower (19.1% vs. 41.2%) and the duration of the second stage of labor was shorter in the study group (47.4 min. vs. 55.6 min.). Episiotomies were less commonly performed (4.7% vs. 8.6%). Second-degree perineal tears were less common in the study group (34.4% vs. 46.4%), higher-order obstetric lacerations occurred more frequently in the study group (2.3% vs. 0.9%). There was no difference in the postpartum hemorrhage rate between the study and control group.

The comparison of newborn data showed a significant difference in the birthweight. Newborns in the study group were on average 95 g heavier, and fourteen (2.3%) newborns (versus four (0.7%) in the control group) had a birthweight ≥4500 g. No difference was present for the components of the neonatal composite outcome and the secondary neonatal outcomes.

Non-inferiority results are summarized in Table 2. Non-inferiority could be confirmed for the predefined outcome parameters.

**Table 2:** Results of the non-inferiority analysis (with non-inferiority margin of −2%) (N = 1224)

	Study group (*n* = 612)	Control group (*n* = 612)	% Difference (95% CI)	*p* value
Cesarean/ instrumental vaginal birth, n (%)	95 (15.5)	110 (18.0)	2.45 (−1.34–6.25)	0.010
Composite outcome^a^, n (%)	14 (2.3)	22 (3.7)	1.34 (−0.65–3.40)	< 0.001

*CI* confidence interval

^a^Newborns with umbilical cord arterial pH < 7.10 and/or 5-min APGAR < 7 and/or transfer to specialist neonatal care; pairs with missing pH values were excluded

The generalized linear mixed effects model did not reveal significant effects of AMU care, BMI or birthweight on the mode of birth (CS plus instrumental birth versus spontaneous birth), nulliparity and a higher age however showed significantly higher odds for CS/vaginal instrumental birth (Model 1, Table 3). Further, AMU care, nulliparity, age, BMI, mode of birth and birthweight did not affect the composite neonatal outcome, see Model 2 in Table 3.

**Table 3:** Results of the generalized linear mixed-effects models for the mode of birth (model 1) and the composite outcome (model 2^Ϯ^)

	Model 1		Model 2^b^	
	aOR (95% CI)	*p* value	aOR (95% CI)	*p* value
Study group	0.86 (0.59–1.26)	0.449	0.59 (0.27–1.26)	0.171
Nulliparous	10.82 (6.55–17.89)	< 0.001	1.47 (0.66–3.24)	0.344
Age (years)				
≤ 29	1.00 (−)		1.00 (−)	
30–34	1.33 (0.84–2.09)	0.220	1.02 (0.42–2.51)	0.960
≥ 35	2.56 (1.56–4.20)	< 0.001	1.05 (0.39–2.79)	0.927
BMI (kg/m^2^) < 25	1.38 (0.93–2.04)	0.115	0.97 (0.45–2.08)	0.935
Birthweight (100 g)	1.04 (0.99–1.08)	0.090	1.04 (0.96–1.14)	0.326
Cesarean/ instrumental vaginal birth	–	–	1.50 (0.61–3.66)	0.376

*aOR* Adjusted Odds Ratio; *CI* Confidence interval; *BMI* Body mass index

^b^Pairs with missing pH values were excluded

Indications for CS and instrumental vaginal birth are listed in Table 4. The leading cause in the study and control group consisted of fetal heart rate abnormalities, followed by delayed second stage of labor.

**Table 4:** Medical indications for instrumental vaginal birth or emergency cesarean by study and control group (*N* = 205)

	Study group (*n* = 95)	Control group (*n* = 110)	*p* value
Indications, n (%)			0.930
Abnormal FHR	41 (43.2)	54 (49.1)	
Delayed second stage	36 (37.9)	29 (26.4)	
Delayed first stage	9 (9.5)	8 (7.3)	
Other	9 (9.5)	19 (17.3)	

*FHR* Fetal heart rate

(b) Causes and outcome of births transferred to standard obstetric care.

Overall, 308 women (50.3%) were transferred from care in the AMU to standard obstetric care. Transfer causes according to their frequency were as follows: request for regional analgesia (28.9%); fetal heart rate abnormalities or stained amniotic fluid (24.4%); pregnancy-induced hypertension / preeclampsia, postdate, or prelabor rupture of membranes requiring induction of labor (19.2%); incomplete placenta, postpartum hemorrhage, or higher-degree laceration (13.6%); and delayed first or second stage of labor (13.3%).

The vaginal birth rate after transfer was 81.5%, including 12.3% instrumental vaginal deliveries. There was no difference in the indications for instrumental vaginal birth and CS between the women after transfer and the control group (data not shown); likewise, postpartum hemorrhage rates were comparable. Of the 13 higher-degree tears in the study group, four occurred during care in the AMU and resulted in transfer to standard obstetric care; the remaining nine tears occurred after transfer to standard obstetric care, see Table 5. Five higher-degree lacerations (38.5%) were associated with instrumental vaginal delivery.

**Table 5:** Higher order obstetric injuries by study and control group and actual place of birth (*N* = 18)

	Study group (*n* = 13)		Control group (*n* = 5)
	During care in AMU (*n* = 4)	After transfer to standard obstetric care (*n* = 9)	
Obstetric injury, n (%)			
Third degree tear	4 (100.0)	7 (77.8)	1 (20.0)
Fourth degree tear	0 (0.0)	2 (22.2)	0 (0.0)
Cervical tear	0 (0.0)	0 (0.0)	4 (80.0)

*AMU* Alongside midwifery units

**Fig. 1:**
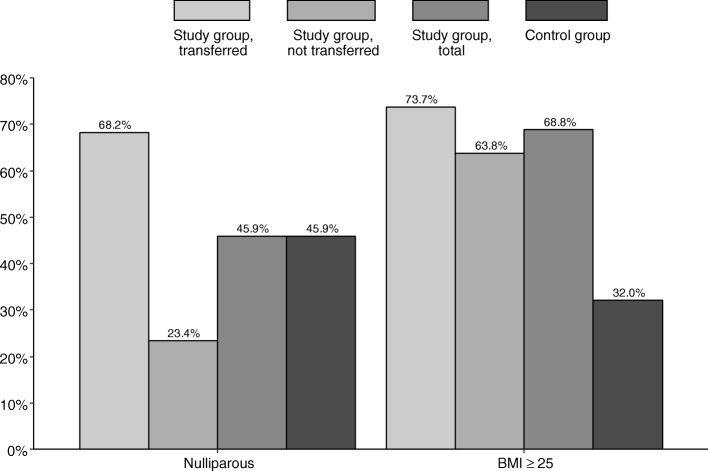
Parity and BMI by study group, transferred (*n* = 308); study group, not transferred (*n* = 304); study group, total (*n* = 612); and control group (*n* = 612)

**Fig. 2:**
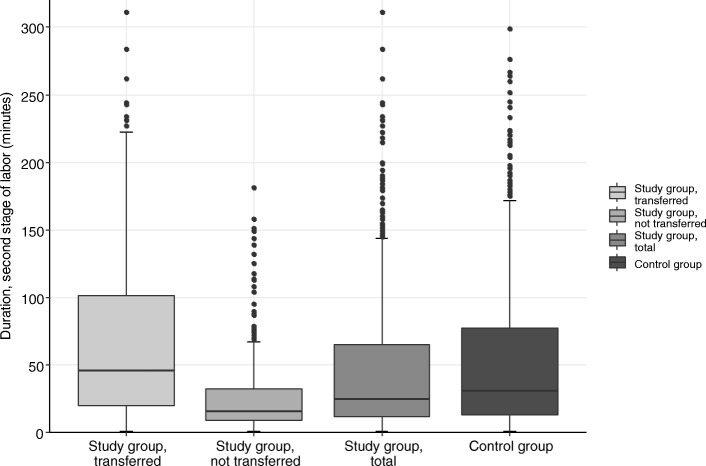
Duration of the second stage of labor (minutes) by study group, transferred (*n* = 251); study group, not transferred (*n* = 303); study group, total (*n* = 554); and control group (*n* = 559)

**Fig. 3:**
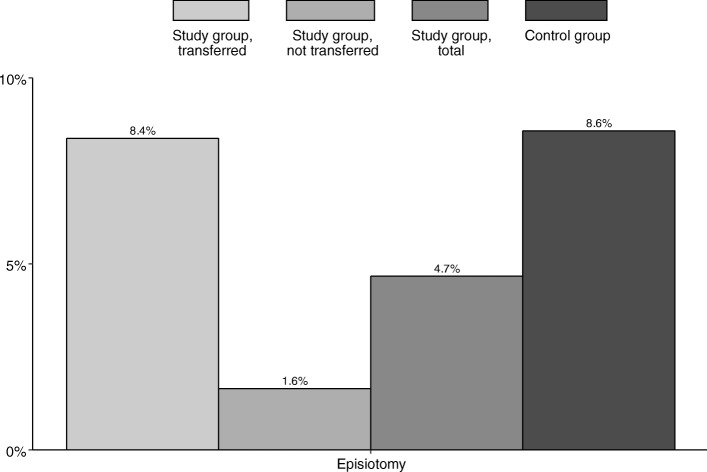
Episiotomy by study group, transferred (*n* = 251); study group, not transferred (*n* = 304); study group, total (*n* = 554); and control group (*n* = 560)

Transfer to standard obstetric care was common in nulliparous women (74.7% of all nulliparous women, representing 68.2% of all transfers), see Fig. 1. 73.7% of transferred women had a BMI ≥ 25 kg/m^2^, see Fig. 1. Second stage of labor was longer after transfer to standard obstetric care, see Fig. 2 (median 46 min, compared to 16 min in the study group, and 31 min in the control group, respectively). Epidural analgesia rates were comparable for the transferred and the control group (38.0% vs. 41.2%). Episiotomies were more frequently performed after transfer to standard obstetric care, see Fig. 3.

**Fig. 4:**
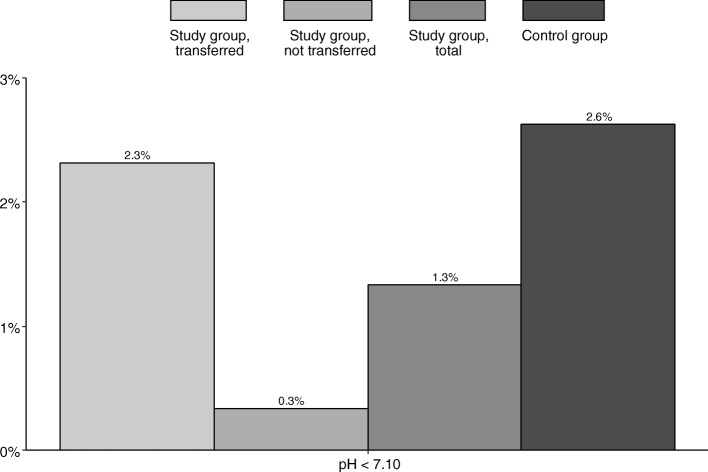
Umbilical cord arterial pH value by study group, transferred (*n* = 302); study group, not transferred (*n* = 298); study group, total (*n* = 600); and control group (*n* = 609)

The number of newborns with an umbilical artery pH < 7.10 was similar in the transferred group and in the newborns of the control group (see Fig. 4), as was the rate of the other neonatal outcome parameters 5-min Apgar score < 7 and need for specialist neonatal care (data not shown).

Logistic regression analysis revealed significant effects of parity, age and birthweight with higher odds for transfer for nulliparous women, higher age and increased birthweight, see Table 6.

**Table 6:** Results of multiple logistic regression analysis for the transfer to obstetric care during or immediately after birth (*N* = 612)

	aOR (95% CI)	*p* value
Nulliparous	8.70 (5.84–13.20)	< 0.001
Age (years)		
≤ 29	1.00 (−)	
30–34	1.26 (0.77–2.05)	0.361
≥ 35	2.12 (1.25–3.65)	0.006
BMI (kg/m^2^) < 25	0.75 (0.51–1.12)	0.159
Birthweight (100 g)	1.05 (1.00–1.09)	0.035

*aOR* Adjusted Odds Ratio; *CI* Confidence interval; *BMI* Body mass index

## Discussion

Our retrospective analysis of births planned in the AMU at our institution, a level three university hospital, confirms the non-inferiority of this model compared to standard obstetric care for selected maternal and newborn outcomes. Additionally, the outcome of women transferred during or immediately after labor serves as an indirect confirmation of the medical safety of this model of care and the appropriateness of the transfer checklist. Our findings apply to healthy women entering labor after uneventful pregnancy. To the best of our knowledge, this is the first study reporting on obstetric and perinatal outcomes of women intending to give birth in an AMU in Germany.

(a) Comparison between the study and control group.

The comparison between the study and control group revealed a non-significant trend towards higher spontaneous and lower instrumental vaginal births. In both groups, the CS rate was below 10%, illustrating the low risk of our cohort.

We analyzed epidural anesthesia and episiotomy rates. Both interventions were less commonly performed in the study group. This finding is in accordance with the existing literature [3, 4].

Causes for the difference in the rate of higher degree perineal lacerations may include obstetric and newborn factors: More women in the study group were overweight or obese, and more than one third of the injuries (38.5%) that occurred after transfer to standard obstetric care were associated with instrumental vaginal births. Additionally, newborns in the study group had a higher mean birthweight, and newborns with birthweight ≥4500 g belonged almost exclusively to the study group (91.7%). Instrumental births and macrosomia are established risk factors for higher-order obstetric lacerations.

Severe neonatal morbidity or even mortality are rare events in high-income countries. For example, severe metabolic acidosis, defined as umbilical cord arterial pH < 7.00 in singleton term newborns occurred with an incidence of 0.21% in Germany in 2017 [9]. The size of our study group was therefore insufficient for a valid comparison of the perinatal outcome in the two models of care. Using a composite outcome, we showed non-inferiority of AMU to standard obstetric care in order to compensate for this shortcoming.

(b) Causes for and outcomes of transferred births.

With 50.3% of women being transferred to standard obstetric care during or immediately after birth, our transfer rate was high. Explanations include the high rate of nulliparous women, and the strict transfer criteria. Nulliparity, higher maternal age and birthweight increased the chance of transfer to standard obstetric care. Higher transfer rates of nulliparous women have also been described by the authors of the Birthplace in England Study (36–45% in nulliparous vs. 9–13% in parous women) [6]. The effect of parity may also explain the low transfer rate (7.0%) of a recently published Austrian study. Here, only 27% of women were nulliparous [10].

The composition of the three groups (study group, transferred; study group, not transferred; and control group) does not allow for a quantitative comparison of outcomes. We therefore limited our analysis to a descriptive presentation. Overall, primary and secondary maternal and newborn outcomes were comparable between the transferred and the control group. We take this result as indirect proof for the safety of births in our AMU. Additionally, the comparability of maternal and newborn outcomes in the transferred group and the control group may serve as evidence for the adequacy of the transfer checklist.

Various factors limit a direct comparison of our results with other studies. These include differences in study design and methods, e.g. with respect to randomization, analysis according to intended versus actual place of birth, and risk assessment. Furthermore, variations exist in birth settings, since organization of maternal healthcare provision is country-specific; this pertains to the key provider of care during birth (midwife versus general practitioner versus specialist); the location of alongside or freestanding midwifery units with respect to the obstetric unit; and the transfer modalities to obstetric units in case of abnormalities occurring during or after labor.

In the systematic reviews by Bohren et al. 2017 [3] and Sandall et al. 2016 [4] only randomized controlled or cluster-randomized trials were included. One-to-one intrapartum support was compared with ‘usual’ care in any setting for its effect on various obstetric outcomes in the former, midwife-led continuity of care models versus other models of care in the latter analysis. Among other outcome variables, higher rates of spontaneous births, lower rates of regional analgesia, a shorter duration of labor, higher 5-min Apgar scores, and no difference in the perineal trauma rates were reported in these reviews [3, 4].

Scarf et al. 2018 [11] in their meta-analysis compared maternal and perinatal outcomes by planned place of birth. Twenty-eight publications with different study designs and methods were included, illustrating the above mentioned limitations. The reported outcomes were not stratified according to core obstetric criteria including parity, epidural analgesia, and oxytocin augmentation. The transfer rates and outcomes after transfer were not mentioned, nor the profession of the providers looking after women who gave birth in hospitals [11].

Of all non-randomized studies, the ‘Birthplace in England national prospective cohort study’ represents the largest study of its kind with the most rigorous design [6]. Maternal and perinatal outcomes of 64,538 low-risk women were prospectively analyzed according to the planned place of birth. With the exception of planned home birth in nulliparous women, the perinatal outcome was comparable in midwifery-led models of care compared to births in an obstetric unit, with less maternal interventions in the former group [6].

More recent European studies share our retrospective design, however, study groups or control groups are different. Gidaszewksi et al. 2019 investigated nulliparous women only and compared caseload with standard midwifery-led care [12]. Jepsen et al. 2018 included at-risk women and compared caseload with standard midwifery care [13]. The study design applied by Bartuseviciene et al. 2018 does not allow the calculation of the number of women who actually received standard obstetric care [14]. In the above cited study by Bodner-Adler et al. 2017, the control group was chosen for matching after (successful vaginal) birth, thereby precluding a direct comparison with the study group. Additionally, care in AMU included oxytocin augmentation and i.v.-opioid analgesia [10].

Strengths of our study include the strict inclusion and exclusion criteria applied for both, study and control group; the pre-specified and documented transfer criteria; and the description of the maternal and newborn outcome after transfer from AMU to standard obstetric care.

Limitations of our study pertain to its retrospective design and the size of the study and control group, which precludes the analysis of rare maternal or newborn complications. Additionally, the high transfer rate has to be borne in mind for comparison with other studies.

## Conclusion

Our comparison of the maternal and perinatal outcome of births planned in AMU with standard obstetric care revealed the non-inferiority and safety of the midwife-led model. This beneficial outcome however requires a clear definition of low-risk pregnancy, and strict admission and transfer criteria.

## Data Availability

The datasets used and analysed during the current study are available from the corresponding author on request.

## Abbreviations

AMU: Alongside midwifery unit
BE: Base excess
BMI: Body mass index
CI: Confidence interval
CS: Cesarean section
FHR: Fetal heart rate
FMU: Freestanding midwifery units
i.v.: Intravenous
MLC: Midwife-led care
SD: Standard deviation
